# Heuristic Evaluation of an IoMT System for Remote Health Monitoring in Senior Care

**DOI:** 10.3390/ijerph17051586

**Published:** 2020-03-01

**Authors:** Pedro C. Santana-Mancilla, Luis E. Anido-Rifón, Juan Contreras-Castillo, Raymundo Buenrostro-Mariscal

**Affiliations:** 1 atlanTTic Research Center, School of Telecommunications Engineering, University of Vigo, 36310 Vigo, Spain; psantana@uvigo.es (P.C.S.-M.); lanido@gist.uvigo.es (L.E.A.-R.); 2 School of Telematics, University of Colima, Colima 28040, Mexico; raymundo@ucol.mx

**Keywords:** older adults, health monitoring, Internet of Medical Things, usability, heuristic evaluation, aging

## Abstract

This paper presents the usability assessment of the design of an Internet of Medical Things (IoMT) system for older adults; the evaluation, using heuristics, was held early on the design process to assess potential problems with the system and was found to be an efficient method to find issues with the application design and led to significant usability improvements on the IoMT platform.

## 1. Introduction

The Internet of Things (IoT), defined as the interconnection of any daily use device (or among them) anywhere and anytime to the Internet [1], have penetrated several fields of healthcare [2], ranging from reminders of medication intakes [3] to the remote monitoring of patients’ vital signs [4]. Recent advances in sensors and mobile devices have led to the development of wearable devices that connect with smart phones to analyze the data obtained from people who monitor their health using them [5]. Through IoT medical technology, physicians or caregivers (responsible for the health of patients) can remotely supervise, in real time, the physical condition of patients [6], these advances have led the coining of the term Internet of Medical Things (IoMT), which is customized healthcare IoT [7].

Based on the needs of elderly people caregivers detected in a questionnaire we applied during the course of this research, we present a proposal for a real-time health monitoring IoMT system focused on supervising older adults living in geriatric residences, aimed to provide physicians and caregivers in nursing homes with a support tool.

Usability allows us to evaluate how easy it is to use a system, as well as how functionality is related to both the task and the person performing it [8]; for example, there are system’s elements perceived as very useful for a particular group of users, but for another, their use could be viewed as a catastrophe.

There are multiple tests to measure usability, one of them is the Heuristic Evaluation, which consists of an inspection evaluation that should be executed, preferably by expert evaluators based on previously established heuristic principles [9], used to catalog the findings according to the degree of the detected problem. An inspection usability evaluation consists of having evaluators inspecting the interface to find usability problems in a design [10].

The objective of this article is to present the procedure and the results of an IoMT-system early-stage heuristic evaluation of usability to determine its friendliness level for caregivers and family members.

## 2. Related Work

This section presents a comparison of different approaches, design context and products discussed within the context of providing health monitoring for elders using Internet of Medical Things technologies.

For example, Li et al. [11] introduces a wireless sensor network health monitoring and alarm system that monitors, stores in a database, and analyzes body health data to send warning messages when an abnormal behavior is detected.

Chandel et al. [12] propose the use of sensors embedded in commercial mobile devices for continuously monitoring healthcare or fitness on elderly, due to their attractive form factor and low power consumption.

The system proposed by Roman et al. [13] consists of an early wearable prototype for health monitoring that measures body temperature, heart rate, and fall detection to show these data on a Liquid Cristal Display (LCD) screen and send notifications to the caregiver using a Global System for Mobile communications (GSM) module.

In contrast with those systems, our proposal aimed to create a system that provides complete support for elders and their caregivers on nursing homes; Table 1 shows the advantages of our proposal in relation to the presented related works.

## 3. Methods

This research is using the User Centered Design (UCD) methodology [14], which is an iterative process with four stages: Specify the usage context, Specify requirements, Produce design solutions, and Evaluation.

### 3.1. Specify the Usage Context

To determine the users’ use context, we developed and applied a semi-structured interview (see Appendix A) to 14 caregivers in five elderly residences in the State of Colima in Mexico. We analyzed the recordings using thematic analysis to identify the characteristics that the IoMT system should include to support the healthcare monitoring of the elderly living in nursing homes. The interviews were analyzed through a comparative verification of the evidence using the MAXqda2 software (VERBI Software GmbH, Berlin, Germany) [15]. As a first step, the authors conducted a systematic analysis of the transcripts of the interviews to obtain the categories and subcategories that helped deepen the understanding of the information provided by the interviewees. Subsequently, the data was coded and analyzed through an interpretation of what the interviewees said.

Some of the most relevant findings were:

- *Remote Monitoring*: Have real-time internet-based remote monitoring of elderly’s vital signs to create a digital history of these records.
- *Alert Notifications*: Generate and set alerts for caregivers and family members when the IoMT system detects an unusual situation.
- *Medical Record*: The system must be able to store the health records and offer a simple and secure access to them in real time.
- *Communication with Family*: To increase the family members’ involvement in the elderly care, the IoMT system must have a function to send detailed reports of elders’ activity to the family.

Based on those findings, the next UCD phase started.

### 3.2. Specify Requirements

#### 3.2.1. System Architecture

**Figure 1:**
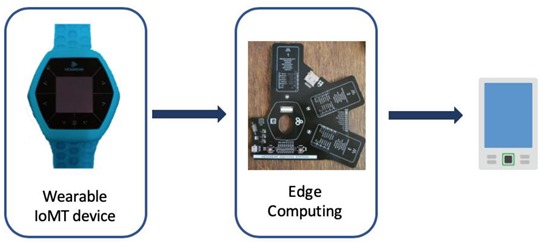
Internet of Medical Things (IoMT) system architecture.

Figure 1 illustrates the IoMT system architecture.

The authors used the Hexiwear biometric bracelet as a wearable IoMT device for the remote monitoring and measure of heart rate, temperature, and calories of the patients wearing them [16].

While the Hexiwear bracelet includes its cloud platform called “WolkAbout IoT Platform”, which stores all the sensed information and makes it available to developers through its application programming interface (API), it is not the only available option.

Instead of using traditional computational equipment (such as a PC) to transfer the information from sensors and send the data to the cloud to process them, the use of microcontrollers such as Arduino or RaspberryPi is spreading rapidly. They have enough computing power and memory onboard to perform lightweight tasks, information processing, and direct transmission to the cloud (with an available internet connection). This paradigm is known as Edge Computing [17], since it allows analyzing relevant data almost in real-time near where the generation of the data occurs, at the edge of the internet. To read sensor data from the edge layer and send it to the smartphone, we utilized the Bluetooth Smart (BLE) through Javascript [18].

#### 3.2.2. Software Requirements

The software functional requirements (which are considered the cornerstone in a software development project) are conditions or capabilities that must be included in the application, requested by a customer or obtained from the users’ context to solve a problem or achieve an objective [19].

Table 2 and Table 3 list the requirements that are directly linked to the IoMT app and describe its complete functionality, as well as the conditions that must be met, obtained from the interviews and the analysis of the project context.

Non-functional requirements are the requirements that express conditions that the software must meet or specific qualities of the software [19].

Table 3 presents the non-functional requirements for the IoMT app.

### 3.3. Produce Design Solutions

**Figure 2:**
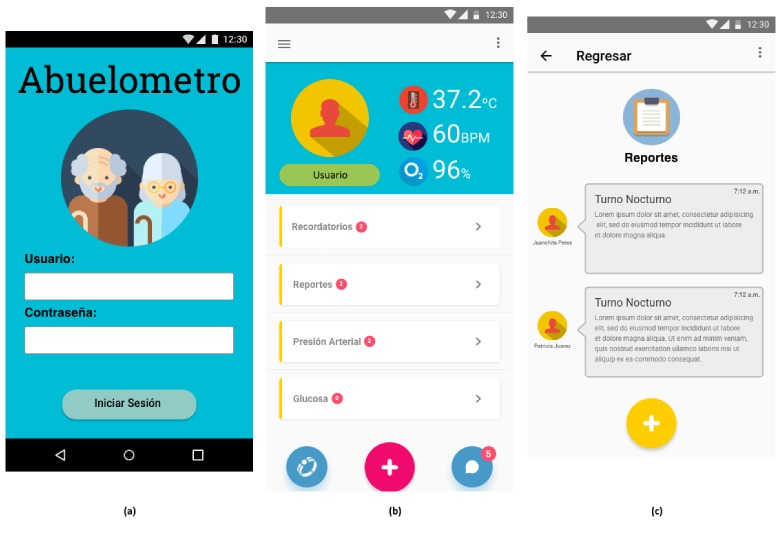
(**a**) Screen for login; (**b**) main screen with monitoring data and notifications; and (**c**) communication screen.

With the requirements and the architecture, we created a medium-fidelity prototype of the mobile application with a mockup software. Figure 2 shows the main wireframes of the prototype.

### 3.4. Evaluation

At this stage of the project, we performed a heuristic evaluation study through an expert review of the IoMT prototype to ensure that the requirements identified after the contextual study were included in an easy-to-use manner for caregivers and family members and before the development of a fully functional application.

#### 3.4.1. Participants

A pilot test with the IoMT prototype was performed, first, by a single expert and later by five different experts. The six evaluators (five men and one woman) belong to the area of computer science and are considered experts in usability and interfaces [20]. The average age of the six experts was 36 years (min 28, max 55), and the general information of the evaluators is shown in Table 4.

The result of the heuristic evaluation is a list of the usability problems with their respective degrees of severity, from which the interface designers can prioritize and make the necessary corrections.

#### 3.4.2. Process

Before each evaluation, the heuristics and the severity scale of the problems were discussed briefly with the evaluators, to make sure everything was clear. Each of the evaluators of the IoMT system performed the evaluation individually to avoid biases and ensure independent results.

The evaluated heuristics principles were: (1) Visibility of system status; (2) Match between system and the real world; (3) User control and freedom; (4) Consistency and standards; (5) Error prevention; (6) Recognition rather than recall; (7) Flexibility and efficiency of use; (8) Aesthetic and minimalist design; (9) Help users recognize, diagnose, and recover from errors; (10) Help and documentation. According to Nielsen [21], the values for the ratings were between 0 (Not a problem) and 5 (Catastrophe).

Each expert had an evaluation form that contained the 10 heuristics and the description of the possible problems to be found, each problem or item had a scale to evaluate its severity.

## 4. Results

The experts identified a total of 47 problems in the IoMT system of which 18 (38.29%) were unique problems.

Table 5 shows experts’ classification of the problem’s severity. About 80.85% of the problems were cataloged as “Can be improved”. Expert 6 identified a critical problem and no evaluator identified problems as catastrophe.

No problem was identified at the same time by all the evaluators, 18 items (38.29%) were identified as problems only by an expert. All the problems identified are distributed in all ten heuristics, that is, a particularly problematic heuristic was not found. It is important to point out that 17 items (36.17%) were not identified as problems.

**Figure 3:**
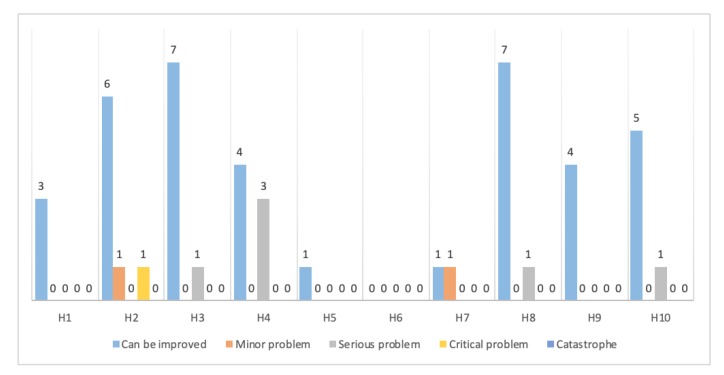
Problems identified in each heuristic classified according to their degree of severity.

Figure 3 illustrates the relationship between the heuristics and the severity of the problems found by the experts. The most frequent classification was “Can be improved” in which 38 (80.8%) of the problems were identified and “Serious problem” with 6 (12.7%) problems in that category. Two (4.2%) problems were classified as “Minor problem” and one (2.1%) as “Critical problem”.

**Figure 4:**
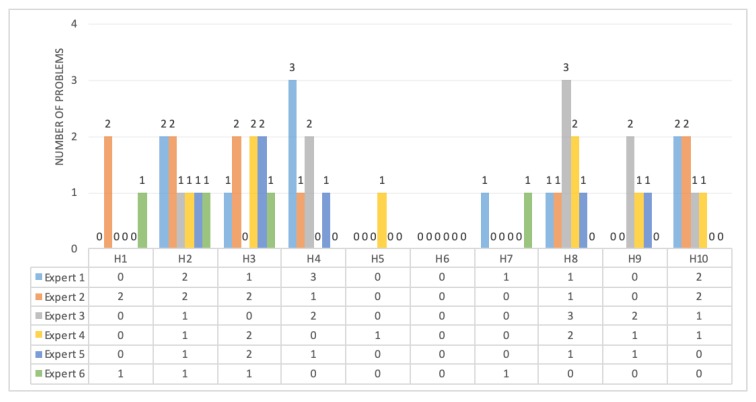
Distribution of problems in heuristics.

Figure 4 shows the distribution of the problems in the heuristics. According to the evaluators, the heuristic “Recognition rather than recall” did not present problems, while the heuristics with more problems were “Match between system and the real world”, “User control and freedom”, and “Aesthetic and minimalist design” (17.02% each), followed by the heuristic “Help and documentation” with 12.76%.

## 5. Discussion

This study confirmed that experts validated a strong level of usability toward our IoMT system for elderly health monitoring. The literature discusses different design concepts and products to improve health monitoring for seniors, mostly living in their own homes. The main contribution of this paper is to validate the feasibility of the use of our IoMT application to support caregiving through continuous health monitoring in a nursing home environment.

The heuristic evaluation proved that it is a feasible method to evaluate the usability of a system based on sensors and mobile devices, so evaluating the IoMT helped in discovering usability problems to be solved before its official release to the elderly and caregivers in public nursing homes. Our results were in accordance with previous studies reported in the literature, such as the case studies presented by Alexandru et al. [22], which consisted of a heuristics evaluation of mobile applications suited for helping people to manage a healthy lifestyle. Furthermore, the work of Stellefson et al. [23] performed a heuristic evaluation of a system designed to help educate seniors with Chronic Obstructive Pulmonary Disease (COPD) to determine whether the prototype was successful in adhering to formal usability guidelines for this population.

## 6. Conclusions

This paper presented the heuristic evaluation of an IoMT platform with a total of 47 problems identified as potential difficulties when used by users.

Even though some usability problems were identified in the heuristic evaluation, this type of evaluation depends on the experience of the evaluators; thus, some problems could only be found by a real caregiver. Therefore, after addressing and resolving these findings, a user assessment with actual caregivers was performed and reported on [6].

## Figures and Tables

**Table 1:** Advantages of related works.

	IoT Health Monitoring	Real Time Warnings	Availability and Low Cost	Non-Invasive
Li et al. [11]	X		X	X
Chandel et al. [12]	X	X		X
Roman et al. [13]	X	X	X	
Our proposal	X	X	X	X

**Table 2:** IoMT app functional requirements.

Functional Requirement	Description
FR-01	The mobile application must have user authentication.
FR-02	The mobile application should be able to show caregivers the latest data recorded by vital signs sensors.
FR-03	The mobile application must allow caregivers to open a communication module to communicate with the elderly relative.
FR-04	The mobile application should allow family members to open a communication module to communicate with caregivers of the elderly.
FR-05	The mobile application should allow the caregiver and the relative to navigate through a calendar, in order to visualize the data generated on the selected days and with the possibility of adding reminders for those days.
FR-06	The mobile application should allow caregivers to add, edit, or delete reports of diseases or illnesses.
FR-07	The mobile application should allow the caregiver to access clinical information of the elderly person as required.
FR-08	The mobile application should allow the relative to review information about the illnesses or diseases of the elderly.

**Table 3:** IoMT app non-functional requirements.

Non-Functional Requirement	Description
NFR-01	The device with the sensors for monitoring vital signs should have Bluetooth Smart (BLE) to send the generated data to the edge microcontroller.
NFR-02	The smartphone must have a version of the Android operating system.
NFR-03	The availability of the system must be permanent (100%), service level for users 24/7.
NFR-04	The stored data can be consulted and updated permanently and simultaneously, without affecting the response time.
NFR-05	The system must have an updateable documentation that allows carrying out maintenance operations with the least possible effort.
NFR-06	The system must support concurrency of users according to the resources of the infrastructure.

**Table 4:** Expert evaluators characteristics.

Expert	Genre	Age
1 (Pilot)	Male	38
2	Male	34
3	Male	32
4	Male	55
5	Male	28
6	Female	33

**Table 5:** Number of problems and their classification by expert.

Classification	E1	E2	E3	E4	E5	E6	Total
Can be improved	6	10	8	7	5	2	38
Minor problem	0	0	0	1	0	1	2
Serious problem	4	0	1	0	1	0	6
Critical problem	0	0	0	0	0	1	1
Catastrophe	0	0	0	0	0	0	0
Total	10	10	9	8	6	4	47

## Appendix A

### Generals

1. What is your name?
2. What is your age?
3. What is your occupation?
4. Do you have a specialty?
5. How long have you worked here?

### Services

6. What services does this nursing home offer?
7. With how many rooms does it count?
8. What is the number of residents staying in this place?
9. What are the requirements to receive the elderly?
10. What type of staff does the nursing home have?
  - Briefly describe the tasks and functions of each worker.

### Contact with Relatives

11. What percentage of the elderly maintain contact with their relatives?
  - How is the interaction of the elderly with the family?
12. Are family members aware of the health of the elderly?
  - How do family members help supervise the health of the elderly?
13. How does the family intervene in case the elderly person needs medical attention?

### Health Monitoring

14. How many people do you have in your care?
15. Do you have a care plan for the elderly?
  - What can the care plan contain?
  - Normally, what is the time interval in which elders undergo regular medical check-ups?
16. During a routine medical diagnosis, what are the physiological parameters that are obtained?
  - How do you measure these values?
  - Have difficulties been encountered in this process? List them.
17. Do you keep a record of the elder’s health status?
  - How do you do that?
  - Are there problems when carrying out the registration? List them.
18. What is the protocol to follow in case the elderly person needs medical attention?
19. What are the main challenges in the health care of an elderly person?
20. What are the needs of the elderly in terms of health?
21. What are the main diseases or conditions that the older adults treated for?
